# Peak Activation Shifts in the Sensorimotor Cortex of Chronic Stroke Patients Following Robot-assisted Rehabilitation Therapy

**DOI:** 10.2174/1874440002114010008

**Published:** 2021-07-07

**Authors:** Loukas G. Astrakas, Shasha Li, Mark P. Ottensmeyer, Christian Pusatere, Michael A. Moskowitz, A. Aria Tzika

**Affiliations:** 1 Medical Physics, Faculty of Medicine, University of Ioannina, Ioannina, Greece; 2 Harvard Medical School, Boston, MA, USA; 3 NMR Surgical Laboratory, Department of Surgery, Center for Surgery, Innovation and Bioengineering, Massachusetts General Hospital, Harvard Medical School, Boston, MA, USA; 4 Athinoula A. Martinos Center of Biomedical Imaging, Department of Radiology, Massachusetts General Hospital, Harvard Medical School, Boston, MA, USA; 5 Medical Device & Simulation Laboratory, Department of Radiology, Massachusetts General Hospital, Boston, MA, USA; 6 Neuroscience Center, Departments of Neurology and Neurosurgery, Massachusetts General Hospital, Boston, MA, USA

**Keywords:** Stroke, Functional magnetic resonance imaging, Hand rehabilitation, MR compatible robotic devices, Brain plasticity, Fugl-meyer upper extremity scale

## Abstract

**Background:**

Ischemic stroke is the most common cause of complex chronic disability and the third leading cause of death worldwide. In recovering stroke patients, peak activation within the ipsilesional primary motor cortex (M1) during the performance of a simple motor task has been shown to exhibit an anterior shift in many studies and a posterior shift in other studies.

**Objective:**

We investigated this discrepancy in chronic stroke patients who completed a robot-assisted rehabilitation therapy program.

**Methods:**

Eight chronic stroke patients with an intact M1 and 13 Healthy Control (HC) volunteers underwent 300 functional magnetic resonance imaging (fMRI) scans while performing a grip task at different force levels with a robotic device. The patients were trained with the same robotic device over a 10-week intervention period and their progress was evaluated serially with the Fugl-Meyer and Modified Ashworth scales. Repeated measure analyses were used to assess group differences in locations of peak activity in the sensorimotor cortex (SM) and the relationship of such changes with scores on the Fugl-Meyer Upper Extremity (FM UE) scale.

**Results:**

Patients moving their stroke-affected hand had proportionally more peak activations in the primary motor area and fewer peak activations in the somatosensory cortex than the healthy controls (P=0.009). They also showed an anterior shift of peak activity on average of 5.3-mm (P<0.001). The shift correlated negatively with FM UE scores (P=0.002).

**Conclusion:**

A stroke rehabilitation grip task with a robotic device was confirmed to be feasible during fMRI scanning and thus amenable to be used to assess plastic changes in neurological motor activity. Location of peak activity in the SM is a promising clinical neuroimaging index for the evaluation and monitoring of chronic stroke patients.

## 1. INTRODUCTION

Ischemic stroke is the most common cause of complex chronic disability and the third leading cause of death worldwide [1]. Although rehabilitation of stroke survivors is generally most effective in the acute stage, especially in the 3-6 week post-stroke time window [2], new rehabilitation strategies offer hope for rehabilitation of chronic stroke patients, even a year or more after a stroke [3]. Notably, a recently developed robot-aided rehabilitation device can be employed for intensive training that complements and improves upon conventional therapy methods [4]. Robotic devices have the advantage of guiding simple, repetitive movements with consistency [5 - 7]. Furthermore, they can be combined with games that keep patients engaged while they are challenged physically [8, 9].

A major challenge that clinicians face in stroke rehabilitation is inter-subject variability in treatment response, which is influenced by numerous factors, including age as well as the severity and location of neurological injury, and the particular behavioral or cognitive deficits that have been generated as a consequence of the injury [10]. Objective evaluation and assessment methods are needed to monitor each individual patient’s recovery and to support personalized rehabilitation planning.

Consistently, neuroimaging studies have demonstrated structural and functional brain changes that reflect plastic remodeling and reorganization of spared areas and pathways [11]. Major neuroimaging findings include the integrity of the cerebrospinal tracts and the appearance of motor-related activation patterns that accompany poststroke neuroplasticity, which has included changes in perilesional, contralesional, non-motor, and secondary motor areas [12]. Based on these findings, biomarkers have been suggested for ischemic stroke diagnosis, treatment staging, and recovery monitoring [13]. Among them, markers involving the ipsilesional primary motor cortex (M1) appear to be particularly important for stroke recovery.

Both animal and human studies have demonstrated a central role of ipsilesional M1 in plasticity that supports stroke recovery, thus making it a primary target for rehabilitation therapy [14]. Successful poststroke recovery has been associated with an activation shift from the contralesional to the ipsilesional sensorimotor cortex (SM) and M1. Exogenous excitation of ipsilesional M1 with transcranial magnetic stimulation has been reported to facilitate improvement in motor function and to enhance motor learning in both acute and chronic stroke patients [15, 16]. Some neuroimaging studies examining the locations and extents of activated areas in the ipsilesional primary SM have reported posterior shifts in activation towards the postcentral gyrus in patients with cortical strokes as well as in patients with subcortical strokes [17 - 22]. However, this displacement appears not to correlate with motor recovery. Thus, its significance in the context of stroke remains elusive. However, it is noteworthy that it has been associated with attention processes and recruitment of primary somatosensory corticospinal fibers [17] as well as increased cortical thickness [21]. Conversely, other studies have reported anterior displacement of SM activation. A study of 8 stroke patients with resultant capsular lesions of the pyramidal tracts, compared to 10 healthy controls (HCs), documented a large ventral extension of the hand field of the contralateral SM in stroke patients [23]. A longitudinal study employing functional imaging with positron emission tomography showed similar findings in two of five patients with subcortical infarcts [24]. Another study with severely affected stroke patients found that recovery was associated with the activation of more anterior premotor pathways [25]. A recent study with chronic stroke patients found activation in the ipsilesional region ventral to the hand area of the primary SM [26]. A popular interpretation of these anterior shifts is that intact premotor cortex (PM) areas may undergo adaptions to compensate for damaged regions [25].

The aim of the present study was to investigate the aforementioned directional discrepancy (posterior *vs*. anterior) of ipsilesional SM displacement in stroke patients. To this end, we performed a longitudinal functional magnetic resonance imaging (fMRI) study following the robot-assisted rehabilitation therapy of 8 chronic stroke patients and compared the findings with those of 13 age-matched HCs. We assessed our patients with clinical scales to explore the potential of using SM activity displacement as a biomarker of clinical recovery. In contrast with previous studies, the same Magnetic Resonance Imaging (MRI)-compatible robotic device was used both for training and during the MRI paradigm, allowing direct assessment of the brain’s functional changes induced by the rehabilitation protocol.

## 2. MATERIALS AND METHODS

### 2.1. Subjects

Eight stroke patients (49.9 ± 12.7 years old, 4 men, 4 women) and 13 age-matched, right-handed healthy volunteers (55.4 ± 13.1 years old, 5 men, 8 women) participated in this study. They were recruited through registries of stroke survivors who have agreed to be contacted for stroke recovery studies that are maintained at Massachusetts General Hospital. The inclusion criteria for stroke patients were: a) first-ever ischemic stroke incurred in middle cerebral artery territory at least 6 months prior to recruitment; b) acute unilateral loss of hand strength score of <4 on the Medical Research Council scale (0-5, 5 = normal) for ≥48 hours; and c) right-handedness according to the Edinburgh Handedness Inventory. The exclusion criteria were: a) the presence of any hearing, vision, language, or cognitive deficit; b) fMRI contraindications; and c) any disorder that impairs the motor function of the stroke-affected hand.

Institutional review board approval of the study was granted by the Partners Human Research Committee (protocol no. 2005P000570). All participants provided informed consent.

### 2.2. Rehabilitation Protocol

Patients were trained under supervision at home with the third-generation Magnetic Resonance Compatible Hand- Induced Robotic Device [27] (“MR_CHIROD”) coupled with an interactive computer game for 45 minutes per day, 3 days per week, over a 10-week period. Their motor performance was assessed before training (baseline), at approximately monthly intervals during training to monitor progress, and 1 month after completing training (follow-up) to assess persistence over time. The clinical motor scales used were: a) the Fugl-Meyer scale for sensorimotor impairment (Upper Extremity, Wrist, Hand, Coordination, Sensation, Passive Joint Motion, Joint Pain, Total); and b) the Modified Ashworth scale (Elbow, Wrist, Fingers, Thumb) for spasticity. Motor deficits were classified according to Page and colleagues’ recommendations [24].

### 2.3. Imaging

The HCs were submitted to a single scanning session, and stroke patients underwent five scan sessions, concordant with clinical motor assessments, including one at baseline, three during rehabilitation, and one at the 1-month follow-up time point. All brain scans were performed with a 3-T Skyra Siemens full-body scanner equipped with a 32-channel phased-array surface coil.

The imaging protocol was comprised of three sequences. Firstly, for high resolution T1-weighted anatomical imaging, we conducted a magnetization-prepared rapid gradient-echo sequence with a repetition time (TR) of 2,300 ms, an echo time (TE) of 2.53 ms, an inversion time of 900 ms, a field of view (FOV) of 256 mm, a resolution of 1 × 1 × 1 mm^3^, and a PAT factor of 2. Secondly, for field mapping, a double-echo fast gradient echo pulse sequence (TR, 650 ms; TE1, 4.92 ms; TE2, 7.38 ms; FOV, 220 mm; resolution, 2 × 2 × 2 mm^3^) was used. Thirdly, for fMRI, we employed a single shot multi-slice echoplanar imaging pulse sequence (TR, 3,000 ms; TE, 30 ms; FOV, 220 mm; resolution, 2 × 2 × 2 mm^3^; PAT factor, 2; simultaneous mutli-slice shift, 3; 100 dynamic scans; and 4 dummy scans).

### 2.4. Motor Task

The motor task was performed with the MR_CHIROD, which was securely attached to the scanner table next to the subject so that the palm and the digits of the right hand could comfortably operate the handle of the device. The motor paradigm had a classical boxcar design with alternating 21-s rest and action periods. During action periods, the subjects compressed and released the MR_CHIROD synchronously with a visual metronome cue (black circle projected on a white-background screen) that oscillated radially at 0.52 Hz (11 cycles over 21 s). During rest periods, the subjects stared at a black fixation cross on the same white background. The squeezing rate of 0.52 Hz provided a good compromise between adequate motor activation, acceptable head motion, the feasibility of task execution, and whole numbers of both squeeze cycles and TRs.

Before scanning, each subject’s maximum grip strength was evaluated with the MR_CHIROD by increasing the grasp-resisting force progressively until a full grasp closure could not be completed. During scanning, the paradigm was executed for both hands using a resistive force equal to 60%, 40%, and 20% of the subject’s maximum grip strength, resulting in six different motor sessions. Subjects rested between sessions to minimize fatigue. Particular care was taken to restrict motion artifacts, including the placement of foam rubber pads with straps across the forehead, arms, and elbows to minimize motion and dampen motion coupling between the subject’s arm and body. The non-moving hand was closely monitored for mirror motions.

### 2.5. Data Analysis

The data were analyzed using the standard statistical parametric mapping toolbox pipeline in SPM12. Preprocessing steps included distortion corrections with FieldMap tools, motion correction, time slicing, normalization to Montreal Neurological Institute (MNI) standard space using registered three-dimensional T1-weighted images and smoothing with a Gaussian kernel of 8 × 8 × 8 mm. Multiple linear regressions were fitted to preprocessed voxel-level data according to a least-squares approach. The dependent variables were the boxcar input function convoluted with a hemodynamic response function and motion regressors. A first-level analysis provided an SPM (T)-map of activated areas for each subject. The peak activation was defined as the voxel with the highest SPM (T)-map value per patient, task, and site. Peak activation represents the most likely position where task-specific functional tissue can be found. Therefore, this metric is commonly used in functional MR imaging somatotopic studies to quantify localization differences. The MNI coordinates of the maximum activation in the combined area of SM and premotor cortex were recorded, and the Human Motor Area Template was used for labeling [28]. A chi-squared test was used to assess group differences in template labels at peak activation. Chi-squared values are reported with degrees of freedom (df). Mean values are reported with standard deviations (SDs).

Mixed linear regression models with coordinates of maximum SM activation as dependent variables and group, age, gender, and force level as covariates were used to assess inter-subject statistical inferences because they can affect repeated measures and correlations in the data of each subject. Mean differences were assessed between groups (patients *vs*. HCs), and between force levels within each group. Mixed linear regression modeling was also used to assess associations of peak activation coordinates with time after the first fMRI session and with Fugl-Meyer Upper Extremity (FM UE) scale scores, which have been established as a reliable measure of hand impairment in stroke patients. Statistical analyses were performed in SPSS version 23.0 (IBM, Inc.). The two-tailed threshold of p < 0.05 was regarded as statistically significant.

## 3. RESULTS

Three patients had strokes affecting the postcentral gyrus. One of these three patients also had a stroke in the superior frontal gyrus, while another had one in the supramarginal gyrus. The remaining five patients had striatocapsular infarcts. Four of them had putaminal infarcts, and the fifth had an insular cortex infarct as well as an external capsular infarct. One of the putaminal infarcts extended to the posterior limb of the internal capsule and the other extended to the superior corona radiata. In all patients, M1 was spared.

**Fig. (1).**
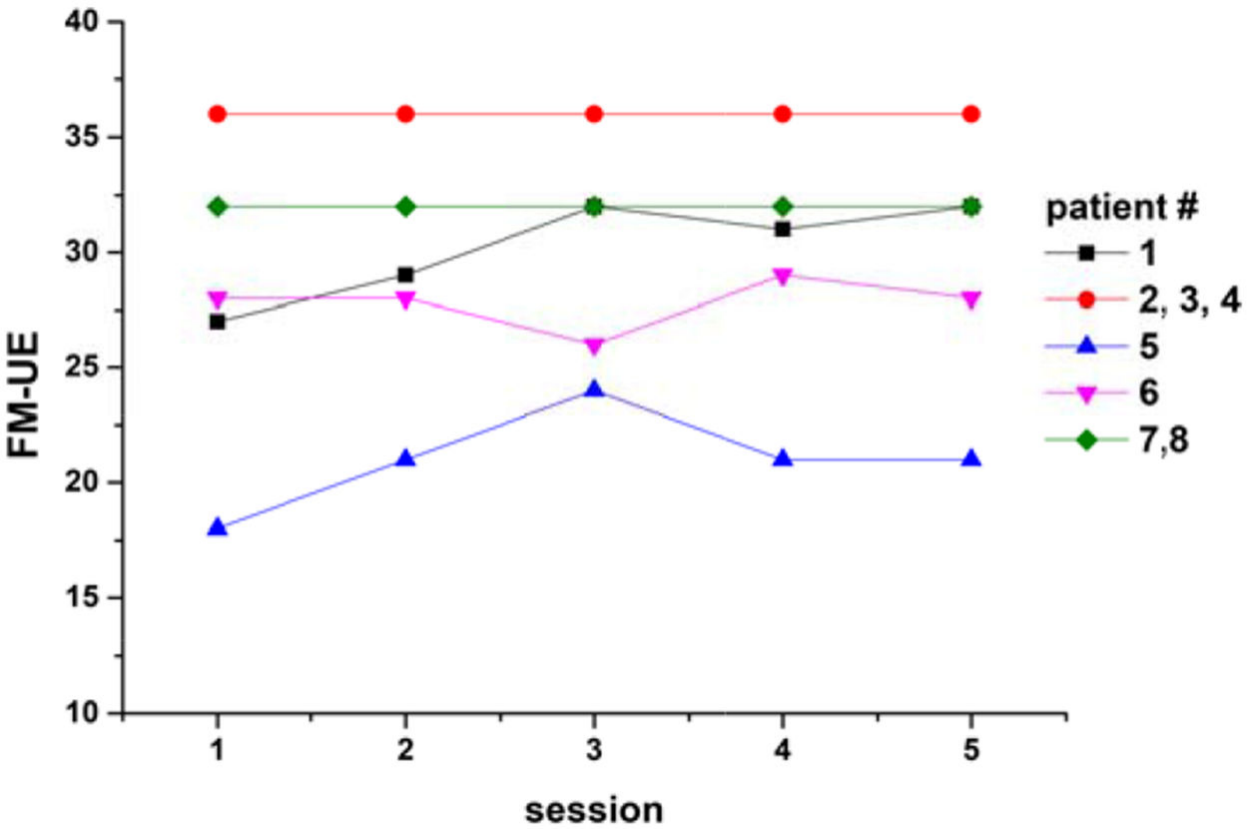
Evolution of the Fugl-Meyer Upper Extremity scale (FM UE) scores over the rehabilitation study period (sessions 1-4) and at 1-month follow-up (session 5) by the patient.

All patients completed the interventional rehabilitation program, except one who dropped out after the second session. Their clinical scores indicate that they all had clinically significant right-sided motor impairment. Specifically, they had a mean FM UE score (±SD) of 21.0 ± 4.4; six were classified as having a moderate impairment and two were classified as having severe impairment. One patient exhibited spasticity (Modified Ashworth scale scores: elbow, 3; wrist, 4; fingers, 3; thumb, 3). According to the FM UE scale, only two patients exhibited marginal progress (+4, +5) during rehabilitation (Fig. 1) [29].

Of 300 scans, 9 were rejected due to excessive motion. For all subjects, the fMRI analysis showed activation in the SM contralateral to the moving hand. As shown in Table 1, according to Human Motor Area Template labeling, stroke patients gripping with their right hands had proportionally more peak activations in the PM and M1 areas and fewer peak activations in the somatosensory cortex (SS) (χ^2^ = 13.3, df = 3, *p* = 0.009).

Comparison of the MNI coordinates of the peak activation sites within SM regions between HCs and stroke patients showed that, for the paretic right hand, patients had, on average, a 5.3-mm anterior shift of ipsilesional peak activation compared to HCs (Table 2).

**Fig. (2).**
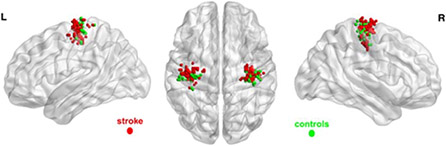
Spatial distributions of peak brain activation during a grip task with both hands. Dots (red, left hemisphere stroke patients; green, healthy control subjects) are overlaid on an International Consortium for Brain Mapping brain surface template (ICBM-152).

For the left non-affected hand, there was no inter-group difference in SM peak activation coordinates. These findings are depicted in Fig. (2), wherein voxels of maximum activation for both hands of subjects in both groups are overlaid on a brain surface template.

Force level did not affect peak activation coordinates in the SM in either group. Time since the first fMRI session did not affect peak activation locations in the stroke patients. Finally, there was a negative association of anterior-posterior coordinates of peak activation with FM UE scores (Table 2), such that the negative sign of association indicates that greater anterior shifts were related to lower clinical scores and thus poorer motor performance. Note that in MNI space, a greater value (or less negative value in this case) in the anterior-posterior axis coordinate represents a shift in the anterior direction. The other directions (right-left, superior-inferior) were not significantly related to clinical scores.

## 4. DISCUSSION

In this case-control longitudinal study, it was shown that functional neuroimaging can be combined with robotic rehabilitation, making a direct assessment of activation in motor areas of the brain during robotic-assisted motor task performance feasible. A force-independent anterior shift of the peak activation in the ipsilesional SM was detected during a simple grip task performed by chronic stroke patients using their paretic hands. Regression analysis revealed a negative association between this shift of SM peak activation and FM UE scores. These findings extend our previous work [30] and corroborate findings by others [31].

As expected, HCs presented a more localized activation pattern than stroke patients within the distributed motor system. The largest activation cluster was consistently within the SM, though the location of peak activation varied (Table 1). In the HC group, the area of peak activation within the SM during gripping was in the SS more often than in the stroke group. Many studies have demonstrated that the SS mediates an important role in motor function [32]. This role is not limited to the processing, translating, and directing of sensory input to the motor system through feedback loops that control, correct, and fine-tune movements [33]. Converging evidence supports the proposed existence of reciprocal, two-way communication between the primary SS and M1, allowing SS not only to control but also to drive movements, even without M1 intervention [34 - 37]. The role of SS becomes prominent in complex movements requiring continued sensory information for precise execution. Although the simple grip motion in our paradigm is not complex, its precise coordination with a visual stimulus may explain the fairly frequent somatosensory overactivation in the HC group.

Stroke patients had peak activation mainly in M1, with large intra-subject variability in the location and intensity of motor activation. A lack of consistency in grip function due to poor motor control is common among stroke patients performing rehabilitation tasks and likely underlies the detected variability in performance. The limited peak activation in SS observed in this study is compatible with an anterior shifting of M1 activation and could be explained in part by the patient cohort composition. Four patients had stroke lesions affecting the postcentral gyrus or had abnormal values on the Fugl-Meyer sensation scale. Animal studies have shown that stroke affecting sensory cortices can cause increased M1 excitability and the formation of a new sensory map that overlaps partly with motor representation [38, 39].

Patients with better motor outcomes had peak activation locations closer to those of HCs. Similar normalization patterns have been documented in studies not employing force-intensive motor tasks. In these studies, a return of normal ipsilesional M1 activation was often found in recovering stroke patients, especially in those showing good recovery, even in the presence of reduced cortical thickness [40, 41].

Although the exact cause of aberrations in M1 ipsilesional activation after stroke is unclear, such aberrations underscore the importance of this area in the recovery process. They may reflect large-scale adaptive alterations of the entire motor network. Indeed, in a study that employed graph analysis of the functional connectome of stroke patients, increased regional centrality of the ipsilesional M1 was observed, indicating an upgraded role of M1 as an information hub in the motor network [42]. When M1 is structurally intact and not completely disconnected, as in our cases, it may recruit previously silent synapses; cortico-cortical pathways might thus recruit intact ventral nonprimary motor areas, while cortico-spinal pathways could compensate for damaged tract fibers, thereby facilitating recovery. Although other factors, such as altered vasomotor activity and neurovascular coupling, cannot be excluded, the hypothesis of unmasking of latent connections after stroke has been supported by many studies and could explain the presently detected shifts in the locations of maximum activation [43]. Enlargement of dendritic fields and modification of synaptic weights by long-term depression and potentiation mechanisms could be the processes responsible for these plastic changes at the neuronal level [44, 45].

Sporadic ventral shifts of brain activity, either to the PM or to the facial motor area, have also been reported in patients who have suffered a stroke affecting M1 [23 - 25]. A meta-analysis of 190 patients executing active movement tasks showed an average 6.5-mm ventral displacement of M1 activity in both the acute and the chronic stroke stage, in agreement with our findings [31]. The same study showed a particularly notable association between ventral shift and motor outcomes among patients who had subcortical strokes. Meanwhile, caudal shifts were detected in patients performing passive tasks, indicating that activity displacement may depend on task demands. The authors, based on previous findings [46], suggested a processing displacement from dorsal Brodmann area (BA)4a, located on the upper and anterior bank of the central sulcus, to ventral BA4p deep in the sulcus as the cause of the ventral shift. Activity in BA4p has been shown to correlate with the magnitude of recovery [47, 48]. While we have found an activation shift of similar size here, our data (Fig. 2) do not support the hypothesis of limited displacement between different M1 segments. We believe that the activation shift varies considerably among stroke patients that should be demonstrable as a statistical effect in large sample population studies or in studies with many scanning sessions like ours.

Although common patterns of brain activity have been observed in stroke patients, each patient presents a unique case. Recovery is a complex dynamic process that depends on numerous genetic, pathophysiological, sociodemographic, and therapeutic factors. Moreover, substantial intra-subject variability of brain imaging has been related to both methodological issues (*i.e.*, the task, data preprocessing and analysis, statistical power, *etc*.) and the instability of the brain’s natural state [49]. The causes of discrepancies between studies reporting posterior shifts in M1 activation and this study are likely multifactorial, including, but not limited to, small cohorts, differences in stroke severity and location, and the use of different motor tasks.

The presently reported correlation between clinical motor scale scores and the detected anterior shift indicates that our approach of performing a rehabilitation task in the scanner is more clinically relevant than assessing activation during simple finger tasks, at least for patients with moderate or severe presentations. The small number of subjects is a limitation of the present study, partially mitigated by the multiple sessions per subject. Another limitation is the lack of temporal analysis of the serial measurements. Our ability to associate brain activity data with the evolution of motor performance was likely limited by the quite limited improvements observed by our patients during the intervention period (Fig. 1. Larger cohorts with more pronounced therapeutic results are needed to elucidate the utility of SM activation shifts in the monitoring or even prediction of rehabilitation outcomes.

Mapping of brain activation during a rehabilitation exercise was a major goal of the present study. Prior studies with similar goals have used either simple motor tasks (*e.g.* finger tapping) or complex movements requiring manual dexterity (*e.g.* finger to thumb opposition task). In stroke cases, simpler tasks may not probe the motor network sufficiently, whereas difficult or complicated tasks performed during fMRI may result in non-compliance and thus poor brain activation. The present study shows that brain activation patterns in force-intensive rehabilitation tasks might not coincide with those produced by finger or hand tasks and might be better for monitoring rehabilitation progress. Grip tasks are feasible for most stroke patients and clinically relevant because they are primary rehabilitation targets for improving function (*e.g.*, supporting and holding objects).

Performing intense tasks during fMRI presents serious challenges, including the need for specialized MRI-compatible training devices and the potential for inducing mirror movements and significant head motion, which themselves may correlate with brain activation and thus render the data useless. Due to these challenges, we did not explore the relationship of the activation intensity or extent with motor outcome in this study. Instead, we focused on the location of peak activation as an index that would be less sensitive to motion artifacts than intensity or extent of brain activity.

## CONCLUSION

A stroke rehabilitation grip task using robotic devices is feasible inside an MRI scanner, allowing assessment of the brain’s motor activity. Comparisons with HCs showed that, in moderate or severe stroke patients with intact M1 performing in force-intensive rehabilitation tasks, there is an anterior shift of peak activation. Contrary to previous studies, this shift associates inversely with the FM UE score. Due to the small number of subjects in our study, more populated studies are required to explore the potential role of this shift as a clinical neuroimaging index relevant to poststroke rehabilitation outcomes.

## Figures and Tables

**Table 1.** Frequency distribution and percentage of peak brain activation by the group during grip task performance with the right hand in the human motor area template.

Group	Unlabeled	M1	Dorsal PM	Primary SS	χ^2^ Statistics
HC	4 (10.3%)	26 (66.7%)	0 (0.0%)	9 (23.1%)	χ^2^ = 13.3, df = 3, *P* = 0.009
Stroke	10 (9.3%)	90 (83.3%)	3 (2.8%)	5 (4.6%)	

HC, healthy controls; M1, primary motor cortex; PM, premotor cortex; SS, somatosensory cortex; df, degrees of freedom.

**Table 2.** Comparison of the MNI coordinates of peak activation in the Sensorimotor cortex (SM) between the right hand of Healthy Controls (HCs) and the (right) paretic hand of stroke patients (Stroke), and the statistical results obtained when peak activation coordinates were used as linear regressors of Fugl-Meyer Upper Extremity scale (FM UE) scores.

Orientation	Peak Activation Sites in SM, Mean EM [95% CI]			Peak Activation Coordinates as Linear Regressors of FM UE Scores	
	HC	Stroke	P	Coefficient [95% CI]	P
Right-left	−37.2 [−39.3, −35.3]	−37.1 [−38.2, −36.1]	0.91	0.12 [−0.02, 0.26]	0.106
Anterior-posterior	−22.7 [−23.9, −21.4]	−17.4 [−18.1, −16.7]	<0.001	−0.18 [−0.29 −0.07]	0.002
Superior-inferior	61.8 [59.3, 64.4]	62.9 [61.4, 64.4]	0.49	0.06 [−0.03, 0.15]	0.201

CI, confidence interval.
